# Repellent efficacy of DEET, MyggA, neem (*Azedirachta indica*) oil and chinaberry (*Melia azedarach*) oil against *Anopheles arabiensis*, the principal malaria vector in Ethiopia

**DOI:** 10.1186/s12936-015-0705-4

**Published:** 2015-05-03

**Authors:** Ephrem Abiy, Teshome Gebre-Michael, Meshesha Balkew, Girmay Medhin

**Affiliations:** The Carter Center Ethiopia, North Wollo Zone Health Department, Malaria and Trachoma project office, PO Box 69, Woldia, Northeast Amhara region, Ethiopia; Aklilu Lemma Institute of Pathobiology, Addis Ababa University, PO Box 1176, Addis Ababa, Ethiopia

**Keywords:** DEET, MyggA, Chinaberry oil, Neem, *Anopheles arabiensis*, Vector, Repellent, Niger seed/noog/

## Abstract

**Background:**

In Ethiopia, *Anopheles arabiensis* is the main vector responsible for the transmission of malaria in the country and its control mainly involves application of indoor residual spraying (IRS) and use of insecticide-treated bed nets (ITNs).

**Objective:**

Although the role of repellents for reducing man-vector contact is documented in the literature, the response of *An. arabiensis* to repellents was not previously evaluated under field conditions in Ethiopia.

**Method:**

The trial was conducted in Sodere village assessing the repellent activities of four repellents, of which, two of them were commercially available DEET (N, N-diethyl-1,3-methylbenzamide) and MyggA (p-methane diol) and the other two were laboratory- produced, 20% neem oil and 20% chinaberry oil. A 6 by 6 Latin square design was employed by involving six volunteers who received rotated treatments of repellents and the Ethiopian Niger seed, noog abyssinia (*Guizotia abyssinia*), and locally called as noog oil (diluents to the two plant oils). Each volunteer also served as control. Volunteers were positioned at a distance of 20–40 m from each other and each was treated with one of the repellents, Niger seed/noog/ oil or untreated. Landing mosquitoes were collected from dusk to down using tests tubes. The tests were done in three replicates.

**Results:**

Both DEET and MyggA provided more than 96% protection. The mean protection time for DEET was 8 hrs while the time for MyggA was 6 hrs. Protection obtained from neem oil and chinaberry oil was almost similar (more than 70%), however, the complete protection time for neem was 3 hrs, while that of chinaberry oil was one hour.

**Conclusion:**

The commercial products and laboratory-produced repellents can be utilized by individuals to avoid contact with *An. arabiensis* in Ethiopia.

## Background

In Ethiopia, malaria is a leading cause of public health problem and impediment to socioeconomic development. *Plasmodium falciparum* and *Plasmodium vivax* are the two most common malaria parasites in Ethiopia, having annual percentage prevalence of 60% and 40%, respectively [1-3].

An important feature in the epidemiology of malaria in Ethiopia is the brevity of transmission season that precludes the development of immunity, favouring periodic epidemics with high mortality [2]. In general, knowing the biology, behaviour, habitat and identification of the vector species helps to decide and implement appropriate vector control methods [4]. Mosquitoes of the *Anopheles gambiae* complex are the most important malaria vectors in the world and are composed of seven sibling species namely, *An. gambiae sensu stricto (s.s.), Anopheles arabiensis, Anopheles bwambae, Anopheles merus*, *Anopheles melas* and *Anopheles quadriannulatus* (species A and B). However, only *An. gambiae* s.s. and *An. arabiensis* have become widely distributed and are most efficient vectors. Moreover, they are most important as far as intensified transmission of malaria is concerned [5,6]. Of the *An. gambiae* complex siblings, *An. arabiensis* and *An. quadriannulatus* sp.B are known to occur in Ethiopia [7] and malaria in Ethiopia is transmitted mainly by *An. arabiensis* [8,9]

Vector control is one of the measures applied to reduce malaria transmission by aiming at reducing breeding and survival of mosquito vectors. The available vector control methods are: chemical, biological, genetic, environmental management, personal protection and integrated vector management.

Although there are many vector control methods, most of them are too expensive, ecologically harmful, and environmentally unsafe or they are practically infeasible and inaccessible to be used in poor countries like Ethiopia. Moreover, insecticide resistance is now a major problem facing malaria vector control programnes in most African countries, including Ethiopia, with most important vector species, showing resistance to one or more of the insecticide classes used in vector control [4,8-10].

There is a need to have an intervention that better avoids such problems. This may include the uses of mosquito repellents which may be commercially available or locally produced by the community itself. Repellents have been used to drive away or repel insects or pests. They may be in the form of smoke, spray or aerosol, oils and body lotions. Aerosols and pump sprays are intended for skin applications and treating cloths while liquid, cream, lotion and spray products enable direct skin application. In Ethiopia, wogert (*Silene macroserene*), kebericho (*Echinops kebericho*), tinjut (*Ostostegia integrifolia*), and woira (*Olea europaea*) have been shown to have repellent effects against *An. arabiensis* under laboratory conditions [11]. Plant parts are burned indoors and the smoke is believed to repel and also knock down mosquitoes. They do have limitations in that they need to be applied frequently (hourly or daily) because of their short residual effect and they might have unpleasant side effects, such as coughing because of irritations from the smoke [12]. Therefore, it is important to know which repellent products can be relied on to provide predictable and prolonged protection from insect bites with out causing side effects on human health. So, rather than burning, repellents applied on the skin are preferable and comfortable for individual use. Moreover, insect repellents may be more economically feasible than other vector control methods and they can substitute or they can be alternatives to chemical control methods, such as space spray, which contaminate the environment and are less economically feasible [13]. Repellents are more recommended for people staying outdoors at night for work or leisure and those working in plantations and may be at risk during daytime. Repellents are also useful in combination with LLITNs by protecting people from the bite of mosquitoes before they retire to bed.

Therefore, a trial was conducted in Sodere village to evaluate the repellent efficacy of one synthetic repellent, DEET and three plant based repellents, Mygg A, 20% neem oil and 20% chinaberry oil against *An. arabiensis*. This research project reported the results obtained from the trial.

## Methods

### Site of trial

**Figure 1:**
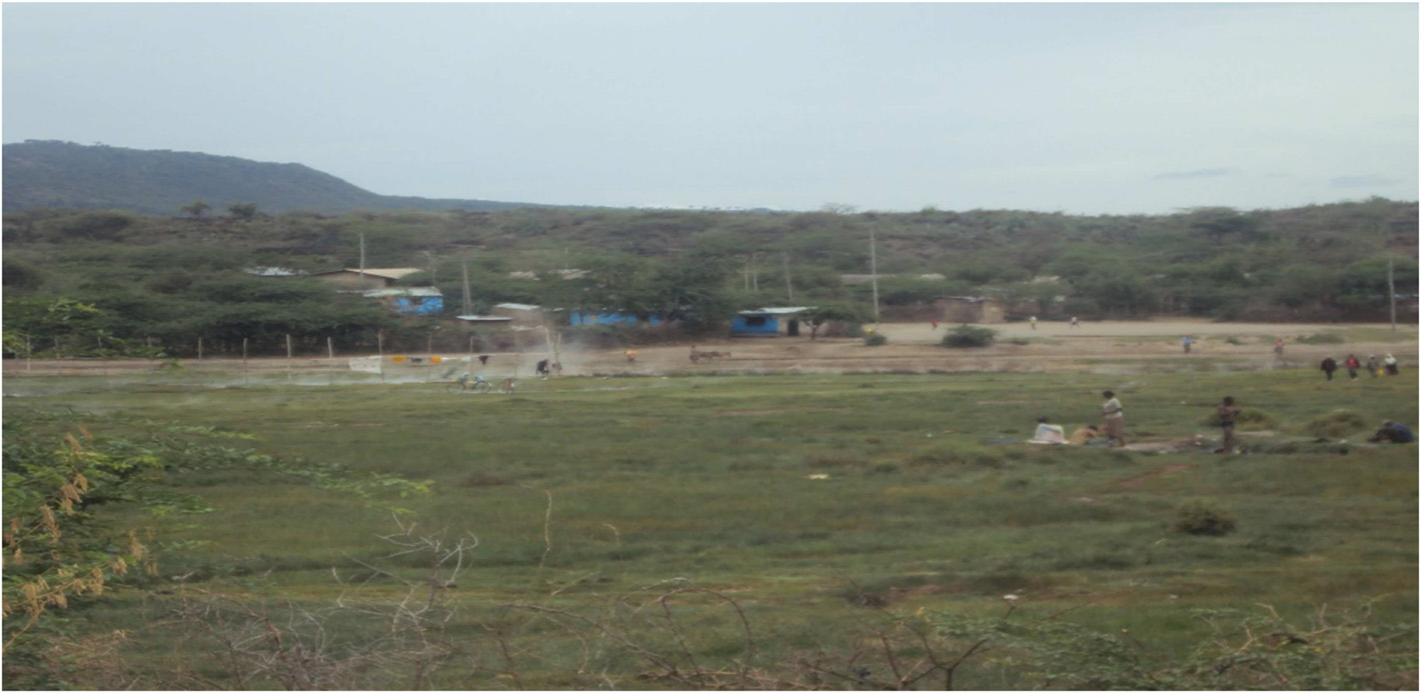
Part of Sodere village.

The study was conducted in Sodere village situated in the Upper Rift Valley from February 2010 to March 2010. It is about 125 km east of Addis Ababa and 25 km south-east of Adama town (see Figure 1). Sodere is known for its recreational area because of its hot spring water for bath and swimming. The hot springs form ideal and abundant breeding sites for *An. arabiensis* throughout the year. The occurrence of *An. arabiensis* was previously reported [9].

### Test products

**Figure 2:**
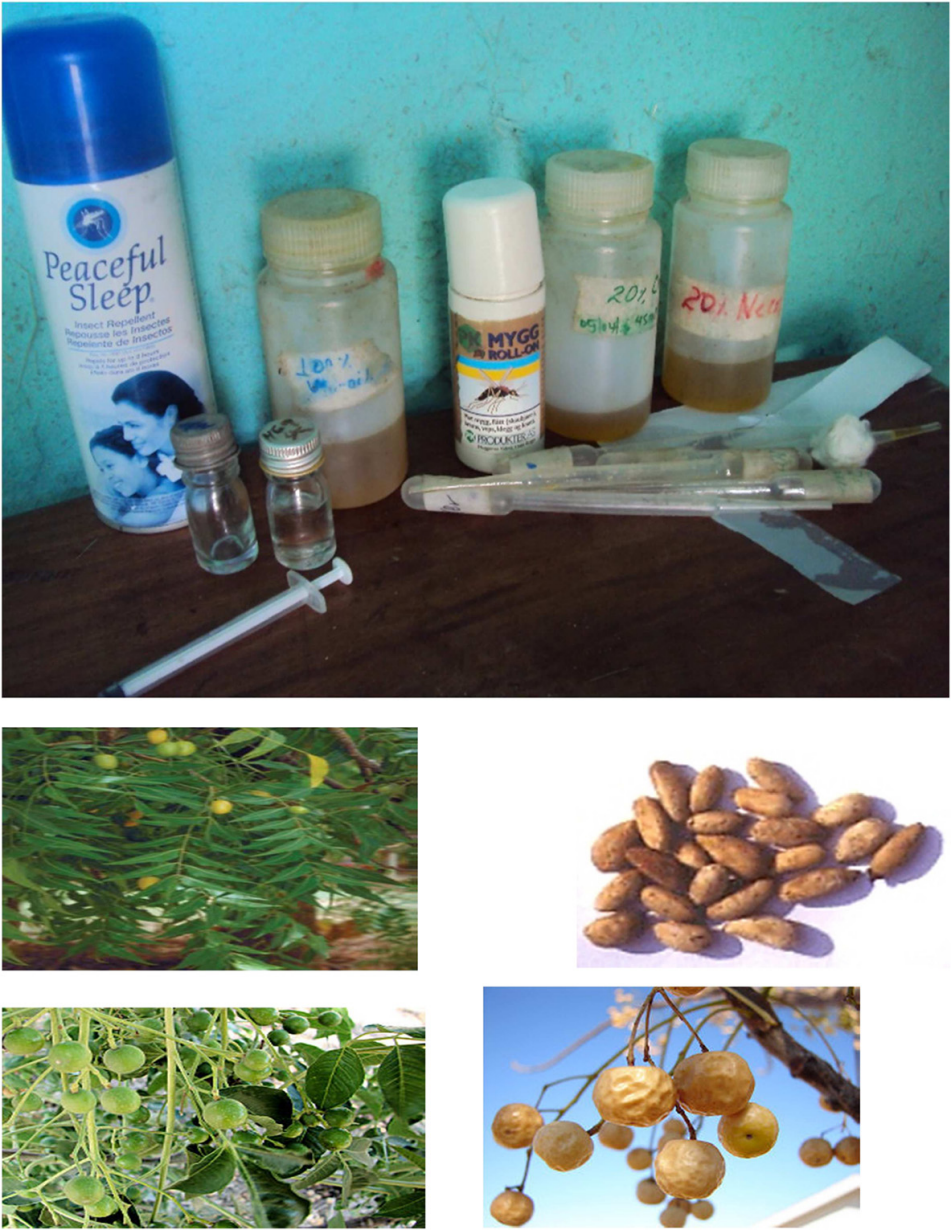
Repellents and equipment used in the test.

The test materials were DEET (N, N-diethyl-1, 3-methylbenzamide), MyggA, neem oil and chinaberry oil. DEET (a.i. 15%; Peaceful–Sleep, Robertsons Home Care (Pty) Ltd., South Africa) was purchased locally from a supermarket. MyggA is a product from Sweden (19% DEET with active perfume of terpene fraction of lavender, geranium and roses). Neem and chinaberry oils were previously hexane extracted at the Ethiopian Public Health Institute/EPHI/ [14] from seeds collected from middle Awash Valley. Edible oil/Niger seed oil/for diluents of neem and chinaberry oils was bought from a super market (See Figure 2).

### Repellent test procedure

The testing methods are based on WHO guide lines [15] for repellent efficacy testing on human skin under field trials. Assessment was made by collecting mosquitoes which land on human volunteers whose legs were treated with repellents and controls. The dose of commercial repellents (DEET (N, N-diethyl-1, 3-methylbenzamide) and MyggA) was based on recommendations of the manufacturers. DEET (N, N-diethyl-1, 3-methylbenzamide) is said to be safe at a concentration of 22-35%, but in the trial 15% was used as per the recommendation of the manufacturer. Neem and chinaberry oils were diluted to 20% using Niger seed (noog abyssinia) oil.

Six volunteers, all of them males with mean age of 22.1 ± 6.9 years, participated in the test. A 6 by 6 Latin-square design was used for the tests. Thus, in the first night of the trial, a volunteer was treated with MyggA, and the second volunteer was treated with 20% neem oil, the third volunteer treated with edible oil, and the fourth volunteer with DEET, the fifth volunteer with 20% chinaberry oil and the sixth volunteer was control without the addition of repellent. On subsequent nights, the treatment application was rotated between the volunteers so that each volunteer was treated with different test repellents.

**Figure 3:**
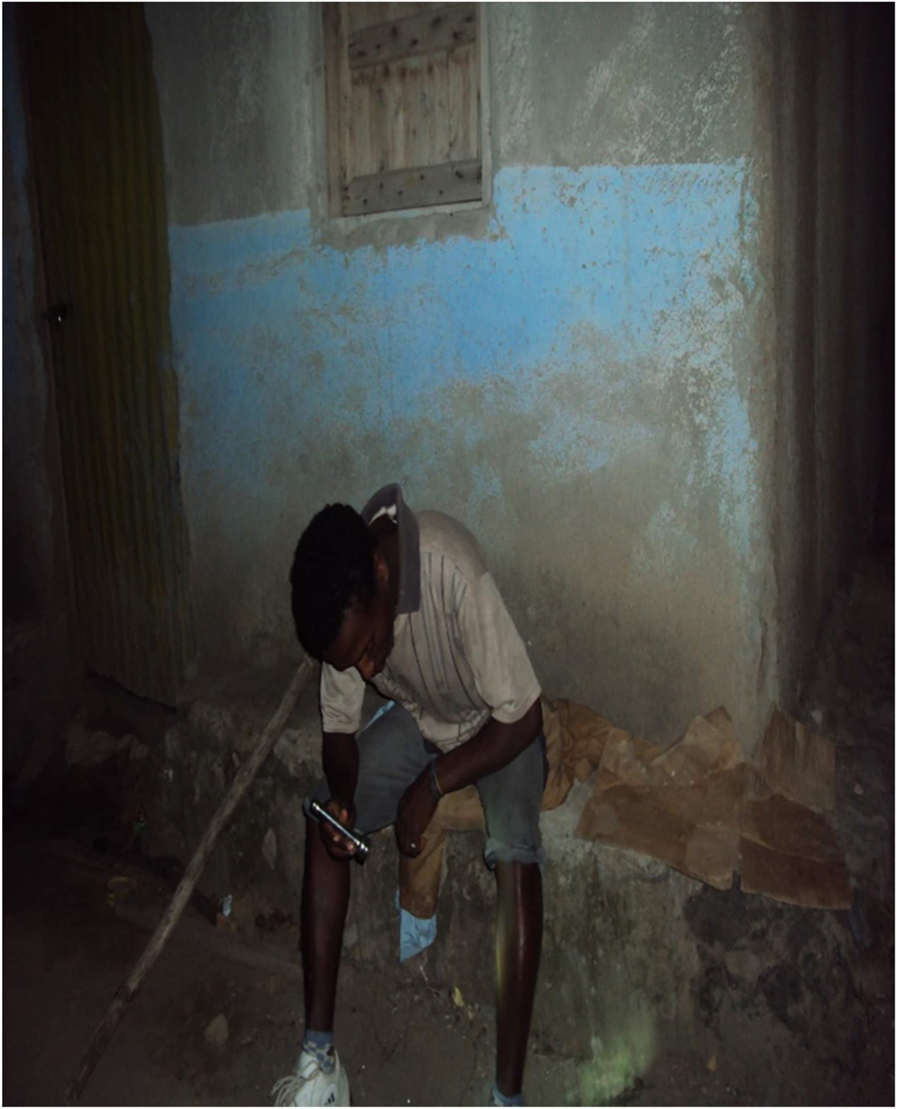
Volunteer collecting mosquitoes.

A volume of 2–3 ml of each of the repellents were applied on the bare legs of volunteers with syringes and spread evenly over the legs from the base of knee to the ankle. After the application of the repellents, volunteers were instructed not to rub, touch, or wet the treated legs (See Figure 3).

Every night volunteers were given test tubes labelled with the kind of treatment and time of mosquito collection. Volunteers were positioned at approximate distance of 20–40 metres from each other and landing mosquito collections took place from 7:00 pm to 6:00 am. A volunteer collected landing mosquitoes from his leg. Mosquitoes were identified in to distinct species based on morphological identification key of [16]. The trial was done in three replicates over a period of one month.

Volunteers were supplied with chloroquine (250 mg) as chemoprophylaxis a week before the start, during and completion of the trial [15].

### Data analysis

Comparison of repellency was made among the six treatment groups using SPSS-Version −13 and the number of *An. arabiensis* collected was subjected to one-way analysis of variance (ANOVA) along with percent protection and complete protection time. Percentage Repellency was calculated as follows:

$$ \mathrm{P}=\left(\left(\mathrm{C}\hbox{-} \mathrm{T}\right)/\mathrm{C}\right)\times 100\% $$

Where *T* is the number of mosquitoes collected from treated volunteers t and *C* is the number of mosquitoes collected from untreated or positive control volunteers [15].

### Ethical considerations

The study obtained ethical clearance from the Institutional Research Board (IRB) of ALIPB. Written consent of volunteers to participate in the trial was also obtained. Volunteers were given chloroquine as prophylaxis one week before the start of the study, every week during the trial and a week after the study was finalized. Treatment was given as per the treatment guideline, 250 mg of tab/kg of body weight (one tablet/week for four weeks).

## Results

Overall, 857 *An. arabiensis* were collected, of which 0.5% were from volunteers treated with DEET, 1.8% from MyggA, 13.12% from 20% neem oil, 14.48% from 20% chinaberry oil, 25.7% from control oil and 44.18% from control without oil (Table 1). Regarding the mean percentage protection, the commercial products performed much better than the laboratory prepared oils giving protection above 95% (Table 1). However, the percentage protection between DEET and MyggA is comparable (p = 0.99). The mean percentage protection of 20% neem oil and 20% chinaberry oil is almost similar (p-value = 1.00). Edible oil has also given 55% protection. The highest mean complete protection time was that of DEET (8 hrs) and the lowest was edible oil (0 hr.). MyggA was able to score a mean complete protection time of 6 hrs.

**Table 1:** **Results of field evaluation of the repellent activities of MyggA, DEET, 20% neem oil and 20% chinaberry oil against** ***An. arabiensis*** **in Sodere, Ethiopia**

**Time (hr)**	**MyggA**	**DEET**	**neem oil**	**chinaberry oil**	**Edible oil**	**Control**
19:00–20:00	0	0	0	1	2	15
20:00–21:00	0	0	0	12	19	35
21:00–22:00	0	0	1	16	27	53
22:00–23:00	0	0	4	23	25	51
23:00–24:00	0	0	30	27	37	57
24:00–1:00	0	0	29	14	51	60
1:00–2:00	6	0	17	6	22	50
2:00–3:00	5	0	9	6	12	22
3:00–4:00	4	2	15	10	10	30
4:00–5:00	0	2	9	2	4	13
5:00–6:00	0	0	1	0	1	10
Total	15	4	115	117	210	396
MPP						
(95% CI)	96 (92–100)	98 (96–100)	71 (58–84)	70 (63–77)	55 (45–65)	0
MCPT	6 hrs	8 hrs	3 hrs	1 hr	0	0

MCPT = Mean Complete Protection Time and MPP = Mean Percent Protection.

**Figure 4:**
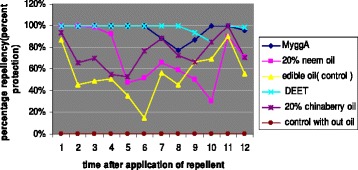
Percent protection provided hourly by Mugger, DEET, 20% neem, 20% chinaberry oil and 2 controls (control oil and control with out oil) against *An. arabiensis* in Sodere, Ethiopia.

Figure 4 depicts percent protection provided by repellents on hourly basis after treatment. The complete protection time of the commercial repellents was different, 8 hrs for DEET and 6 hrs for MyggA. For the first 6 hrs both DEET and MyggA gave 100% protection but after 2 hrs, protection of MyggA was decreased to < 80% while DEET gave 100%. Neem provided 100% protection for 3 hrs and then protection was dropped to 20- 60% after 7 hrs. But, 20% chinaberry gave 90-100% protection for the first 1 hr, and thereafter, dropped to 50-80% after 11 hrs. Although edible oil (control oil), showed percentage repellency of 55 (95% CI = 45 – 65), the mean complete protection time was nil. Mosquito collection was low between 5:00 am and 6:00 am from all treatments including controls.

## Discussion

The use of repellents as personal protective tools to break diseases transmission through avoidance of man-vector contact is a usual way to combat vector-borne diseases [13]. Selection of appropriate repellents together with clothing and other personal protective tools can give adequate protection against bites of mosquitoes.

This study showed that both DEET and MyggA provided the highest comparable protection against *An. arabiensis* and lowest protection from neem and chinaberry oil. Similar studies in Burkina Faso reported 95% repellency and complete protection time of 8 hrs against *An. gambiae s.l.* [17]. Similar observation was also made in Queensland, Australia on different mosquito species [18]. However, a report from Thailand indicated lower repellency than stated above but the mean protection time was similar. This might be due to response variation between mosquito species and the concentration of the active ingredient [19], from Kenya a study has shown that DEET had given 60.2% protection against *An. arabiensis* after 9 hrs of treatment. Recent studies by [20,21] from Australia and at Papua New Guinea showed that 20% DEET gave >95% protection for only 2 hrs against all mosquitoes and overall of 55.8% against *Culex* at the end of 7 hrs of treatment.

In the present study, it was found that the complete protection time of 20% neem oil and 20% chinaberry oil is 3 hrs and 1 hr, respectively in contrast to the report by [22], in which 2% neem provided 12 hrs protection from the bite of mosquitoes.

In India, 2% neem oil diluted with 1-4% coconut oil gave between 80% and 100% protection and four fold protection time than reported here [22-24]. The long protection may be due to effect of the combination neem with the diluents, coconut oil in the Indian test which differs from the present test that used edible oil. The other factor could be variation in the susceptibility status of *Anopheles* species in India (*Anopheles stephensi, Anopheles sundaicus* and *Anopheles fluviatalis)* and *An. arabiensis*.

This study has shown that MyggA provided 96% (92-100%) repellency and 6 hrs complete protection time against *An. arabiensis* in Sodere. There are no documented reports to compare the results. The same is also true for chinaberry oil.

Based on the results of this study, DEET was the most effective repellent against *An. arabiensis* having a higher percent protection of 98% and complete protection time of 8 hr (100% protection till 8 hr). As a result, it can be used and distributed in malaria-endemic areas or wherever there is a high vector capacity to prevent man-vector contact.

## Conclusions

DEET was the most effective repellent against *An. arabiensis* because of its high percent repellency (98%) and complete protection time (8 hr). Mygg A also has shown a high potential of repellency (96%) and protection time (6 hrs) next to DEET. 20% neem oil and 20% chinaberry oil has shown less repellency (71% and 70%, respectively) and complete protection time (3 hrs and 1 hr, respectively) as compared to DEET and MyggA.

### Recommendations

The two commercially available repellents can be used and distributed to malaria endemic areas wherever there is a high biting rate of mosquitoes (high vectorial capacity). The two locally available oils can also be used, but to get several hours of protection applications can be repeated as much as required. In addition to ITNs these repellents can be used for people who work and rest out door.
